# Surgical Considerations for Neoadjuvant Therapy for Pancreatic Adenocarcinoma

**DOI:** 10.3390/cancers15164174

**Published:** 2023-08-19

**Authors:** Anish J. Jain, Jessica E. Maxwell, Matthew H. G. Katz, Rebecca A. Snyder

**Affiliations:** Department of Surgical Oncology, University of Texas MD Anderson Cancer Center, Houston, TX 77030, USA; ajain8@mdanderson.org (A.J.J.);

**Keywords:** pancreatic cancer, pancreatic adenocarcinoma, neoadjuvant therapy, resectability, vascular resection, prehabilitation, CA 19-9 pancreatic cancer, chemotherapy switch

## Abstract

**Simple Summary:**

Pancreatic ductal adenocarcinoma (PDAC) is a morbid malignancy with discouraging survival rates. Enthusiasm for pre-operative therapy (chemotherapy, radiation, chemoradiation) in the treatment of PDAC has grown, with many clinical guidelines recommending its use in patients with borderline resectable tumors. The purpose of this review is to discuss important surgical considerations for the use of pre-operative therapy in patients with pancreatic cancer undergoing curative intent surgery. These considerations include accurately determining tumor resectability, vascular resection, reconstruction when tumors involve surrounding vascular structures, and implementing pre-operative fitness programs to improve treatment outcomes. We also discuss methods to evaluate the response of PDAC to pre-operative therapy such as CA 19-9 levels, imaging studies, and others that are currently being studied for potential use in the future. Preoperative therapy can provide many benefits to patients with pancreatic cancer undergoing surgery, but a comprehensive understanding of several surgical considerations is needed.

**Abstract:**

Pancreatic ductal adenocarcinoma (PDAC) is a challenging disease process with a 5-year survival rate of only 11%. Neoadjuvant therapy in patients with localized pancreatic cancer has multiple theoretical benefits, including improved patient selection for surgery, early delivery of systemic therapy, and assessment of response to therapy. Herein, we review key surgical considerations when selecting patients for neoadjuvant therapy and curative-intent resection. Accurate determination of resectability at diagnosis is critical and should be based on not only anatomic criteria but also biologic and clinical criteria to determine optimal treatment sequencing. Borderline resectable or locally advanced pancreatic cancer is best treated with neoadjuvant therapy and resection, including vascular resection and reconstruction when appropriate. Lastly, providing nutritional, prehabilitation, and supportive care interventions to improve patient fitness prior to surgical intervention and adequately address the adverse effects of therapy is critical.

## 1. Introduction

Recent advances in surgical, systemic, and biologic therapy have significantly improved survival in patients with gastrointestinal cancers. However, pancreatic ductal adenocarcinoma (PDAC) remains a major public health concern, with more than 60,000 cases diagnosed in 2022 and only 11% of patients expected to survive for at least 5 years [1,2]. This is a modest improvement over the 5-year survival rate of 5.3% for PDAC in 2000. This improvement can be attributed to multiple factors: advancements in surgical techniques such as vascular resection and reconstruction, enhanced patient selection for surgery based on holistic criteria, and the increased use of multiagent cytotoxic therapy in the neoadjuvant setting [3].

Researchers pioneered neoadjuvant therapy for PDAC in the early 1990s via several single-institution studies of preoperative chemoradiotherapy (CRT). Those studies demonstrated that CRT could be administered safely in the neoadjuvant setting and provided some benefit based on histopathologic evaluation of resected tumor specimens [4,5,6,7,8,9]. Since that time, enthusiasm for the use of preoperative therapy for borderline resectable PDAC (BR-PDAC) and resectable disease has continued to grow [1].

The rationales for the use of systemic chemotherapy and/or CRT prior to surgery in patients with localized PDAC are well supported. Mouse studies using a Kras-driven PDAC model have demonstrated that pancreatic epithelial cells can be detected in the circulation and liver prior to development of macroscopic pancreatic lesions, supporting the notion that dissemination of PDAC occurs early in tumorigenesis [10]. Preoperative therapy can treat this micrometastatic systemic disease early, which is biologically advantageous. Furthermore, untreated tumor cells can infiltrate the retroperitoneum and neural plexus of the superior mesenteric artery such that even a technically optimal dissection can result in a positive microscopic margin, which can lead to local and distant recurrence [11,12]. Additionally, pancreatectomy is associated with high rates of morbidity and prolonged recovery, both of which can delay or even prevent receipt of adjuvant systemic therapy [13,14,15]. Lastly, administering preoperative therapy allows for improved patient selection for surgery so that only patients most likely to benefit are offered an operation [16]. 

Findings from multi-institutional and international studies have increased support for the use of preoperative therapy of localized PDAC. The recently published long-term results of the PREOPANC-1 trial demonstrated improved survival among patients with resectable PDAC or BR-PDAC treated with preoperative gemcitabine-based CRT compared with upfront resection and adjuvant gemcitabine-based chemotherapy [17]. The ESPAC-5F trial demonstrated similar resection rates among patients with BR-PDAC treated with upfront surgery versus preoperative therapy (FOLFIRINOX, gemcitabine plus capecitabine, or capecitabine-based CRT). The investigators in that trial also noted a significantly increased 12-month disease-free survival rate in patients who received preoperative therapy (59% vs. 33%) [18]. Finally, the recently published Alliance A021501 trial results established the effectiveness of preoperative mFOLFIRINOX in a biologically heterogeneous group of patients with BR-PDAC, with an impressive median survival time of 31 months [19,20]. 

SWOG 1505 was the first prospective trial comparing the efficacy of different systemic chemotherapy regimens in a neoadjuvant setting for resectable PDAC. In that phase 2 study, patients were randomized to either neoadjuvant mFOLFIRINOX or gemcitabine and abraxane followed by resection. Although the study did not demonstrate that neoadjuvant therapy improved overall survival (OS) over that of historical controls, it did demonstrate adequate safety and high resectability rates following preoperative therapy [21]. 

Some patients with BR-PDAC receiving pre-operative therapy will ultimately not undergo curative intent resection. The predominant reason (approximately 25%) is disease progression during said therapy, with other reasons including intolerance of therapy (4%), poor patient conditioning (3%), and loss to follow-up (4%) [22]. However, most studies report upwards of 60% resection rates after administration of pre-operative therapy in patients with BR-PDAC [22,23,24,25].

American Society of Clinical Oncology clinical practice guidelines now recommend neoadjuvant therapy for all patients with BR-PDAC and consideration of neoadjuvant therapy for all patients with resectable PDAC [26]. Additionally, the National Comprehensive Cancer Network (NCCN) recommends neoadjuvant therapy for all patients with BR-PDAC and consideration of preoperative therapy for high-risk patients with resectable PDAC [27]. 

Physicians should incorporate a number of important factors into decision-making when considering the use of preoperative therapy in patients with localized PDAC. These include anatomic resectability, implications of vascular tumor involvement, optimization or prehabilitation of patients undergoing preoperative therapy, and appropriate management of adverse and toxic effects during therapy. 

The focus of this review is to outline these factors and the associated evidence so that all members of a multidisciplinary treatment team appreciate the surgical considerations relevant to treatment sequencing and decision-making for neoadjuvant therapy in PDAC.

## 2. Determination of Resectability

Localized PDAC consists of a spectrum of resectability, specifically anatomically resectable PDAC, BR-PDAC, and locally advanced/unresectable PDAC [3,28]. Resectability is determined based on preoperative diagnostic imaging, specifically, multiphase computed tomography (CT) of the abdomen and pelvis that includes at least an arterial and portal venous phase with thin image slices (<2.5 mm). Additional diagnostic imaging that can inform determination of resectability includes magnetic resonance imaging with contrast and endoscopic ultrasound. Patients who receive preoperative therapy should undergo restaging prior to surgery to assess response to therapy, specifically, the absence of local or distant progression in cross-sectional imaging and biochemical response according to serum CA 19-9 level measurement [27,29].

### 2.1. Anatomic Criteria for Resectability

Most of the current classification systems used to determine tumor resectability are based on anatomic criteria, specifically, the degree of contact between the solid tumor and vasculature. Tumor-vascular contact is categorized as uninvolved, abutment (≤180° circumferential involvement), or encasement (>180° circumferential involvement) [3]. 

Tumors are considered resectable if they have no or minimal (≤180°) contact with mesenteric venous structures (superior mesenteric vein [SMV] and portal vein [PV]) and no contact with arterial structures (superior mesenteric artery [30], celiac axis [CA], and common hepatic artery [CHA]). BR-PDACs have venous involvement (>180°) and/or arterial involvement as noted in Table 1. Locally advanced/unresectable PDAC is considered unresectable at presentation owing to extensive vascular involvement, including arterial encasement (>180°) [3,27]. 

The 2022 NCCN pancreatic cancer treatment guidelines recommend that decisions about resectability be made with a consensus at multidisciplinary meetings or conferences at high-volume centers with access to appropriate high-quality imaging studies to evaluate the extent of the disease. The specific NCCN criteria for resectability (Table 1) are based on anatomic findings from CT imaging at diagnosis [27].

Historically, patients with anatomically resectable PDAC have undergone upfront surgical resection out of concern for possible progression that could preclude future resection. In patients with BR-PDAC or locally advanced/unresectable PDAC, preoperative therapy has been preferred to improve patient selection for surgery and increase the likelihood of margin-negative resection [1,31]. 

Given the precise tumor-vessel relationships that determine anatomic resectability and thus influence treatment options for patients with pancreatic cancer, the use of a structure synoptic template reporting system could be useful. It can ensure that anatomical features of interest are conveyed in a uniform, unambiguous, and concise manner and not omitted from radiologic reports. Additionally, incorporating imaging protocol guidelines in standardized synoptic templates can potentially improve adherence to said guidelines and image quality. In fact, synoptic template reports have been adopted for MRI in rectal cancer staging [32,33,34]. 

An Australian pilot project developed and tested a synoptic report for PDAC derived from international consensus guidelines at two metropolitan pancreatic cancer services to standardize CT reporting in the region. The pilot project was well received by medical staff, whilst highlighting deficiencies in the quality of pancreatic CTs within the community and the hospital systems. Most notably it illustrated the feasibility of a synoptic reporting system for PDAC and supported future implementation of similar efforts [35].

### 2.2. Holistic Criteria for BR-PDAC

Anatomic staging systems do not consider the biologic nature of a patient’s tumor or the physiologic characteristics of individual patients but rather rely solely on the anatomic relationship of the mass and nearby major vasculature. Patients with BR-PDAC can have similar tumor anatomy, but heterogenous physiology and cancer biology [36].

At The University of Texas MD Anderson Cancer Center, we developed a more holistic set of criteria for patients with BR-PDAC that incorporates tumor anatomy (A), tumor biology (B), and a patient’s conditional (C) status [1,16,37]. A report of a study at our institution published in 2008 expanded the definition of BR-PDAC beyond arterial tumor abutment (MD Anderson Type A) to include indeterminate/suspicious extrapancreatic metastatic disease (MD Anderson Type B) and marginal pretreatment performance status (MD Anderson Type C) [16].

We expanded the definition of BR-PDAC based on an increased number of patients with diseases meeting the criteria for MD Anderson Type B or C BR-PDAC. Additionally, we felt that a multifaceted definition of resectability can more accurately estimate the likelihood of margin-negative resection, predict prognosis in surgical patients, and select the optimal treatment sequence. Thus, we proposed resectability criteria that extend beyond just anatomic characteristics (Table 2).

We also hypothesized that the use of the holistic MD Anderson Type A, B, and C criteria for BR-PDAC could predict reasons for deferred resection and postoperative outcomes. In a subsequent retrospective study, we found that metastasis during preoperative therapy ultimately precluded resection in 46% of MD Anderson Type B BR-PDAC patients (those with suspicion of extrapancreatic disease). Similarly, a poor performance status precluded resection in 32% of MD Anderson Type C BR-PDAC patients. However, resected and unresected MD Anderson Type B and C BR-PDAC patients had similar median OS times to patients with resected and unresected clinically resectable PDAC, respectively. Individualizing treatment algorithms based on BR-PDAC type may improve resectability rates and survival [36].

In 2017, the International Association of Pancreatology incorporated these criteria into an international consensus for the definition of BR-PDAC (Table 3). In this definition, BR-PDAC can be classified as BR-A (based on anatomic criteria alone), BR-B (based on biological criteria alone), BR-C (based on conditional criteria alone), or a combination of these criteria (BR-AB, BR-BC, BR-AC, or BR-ABC) [38].

### 2.3. The Role of Laparoscopy

When state-of-the-art CT is available, the routine use of a staging laparoscopy prior to initiation of neoadjuvant treatment may not be easily justified from data in the literature [39]. The NCCN does not consider staging laparoscopy to be a substitute for pre-treatment/pre-operative imaging [27]. Additionally, it is not helpful in determining resectability as tumoral vascular involvement, especially with the SMA, cannot be assessed on laparoscopy. However, the selective use of a diagnostic laparoscopy at the time of planned laparotomy for tumor resection is more appropriate and is likely to be more cost-effective [39,40]. 

Many surgeons advocate the use of diagnostic laparoscopy prior to attempted resection as small liver metastasis and peritoneal disease may be undetectable on even the highest quality axial imaging [41]. A Cochrane database review of the use of diagnostic laparoscopy demonstrated a decreased laparotomy and aborted resection rate from 40% with CT alone to 17% with CT combined with diagnostic laparoscopy [42]. If metastatic disease is detected at diagnostic laparoscopy, patients can avoid the more morbid exploratory laparotomy, experience a more rapid postoperative recovery, and initiate systemic chemotherapy earlier [43,44,45]. Many high-volume institutions, including several NCCN member institutions, routinely employ diagnostic laparoscopy before formal resection to confirm the absence of metastatic disease [27,46]. 

Of note peritoneal washings may be performed as an adjunct to laparoscopy. Positive peritoneal cytology occurs in up to 30% of potentially resectable cases and said patients have similar outcomes to patients with metastatic disease [47,48]. Thus, those patients are not considered candidates for resection, and the NCCN considers positive cytology from peritoneal washings equivalent to M1 disease [27].

## 3. Prognostic Utility of CA 19-9 Levels during Preoperative Therapy for PDAC

As the role of preoperative therapy in the treatment of PDAC expands, the use of reliable assessments of therapy response is important to effectively modify therapeutic regimens and improve patient selection for surgery.

Radiographic imaging, specifically the absence or presence of disease progression, is an important tool for the assessment of response to neoadjuvant therapy. However, in many cases, the primary tumor may remain unchanged on radiographic imaging despite exposure to therapy. Additionally, in the absence of gross progression, radiographic determination of whether occult micrometastases are unresponsive, stable, or responsive to therapy is difficult [49]. 

CA 19-9 is a quantitative biomarker for PDAC that is associated with advanced pathologic stages at high levels. Furthermore, in patients with advanced PDAC who received systemic therapy, alterations in CA 19-9 levels correlated with survival and radiographic response [50,51,52,53]. 

Multiple groups have investigated the prognostic value of changes in CA 19-9 levels by retrospectively analyzing serum CA 19-9 levels before and after neoadjuvant therapy in patients with pancreatic cancer. They all concluded that increased CA 19-9 levels after neoadjuvant therapy are associated with worse survival outcomes [54,55]. Some investigators argued that normalization of CA 19-9 levels after neoadjuvant therapy is the strongest prognostic indicator for long-term survival after resection of PDAC [49,56]. Several other studies demonstrated that a sizeable decrease in CA 19-9 level, ranging from more than 30% to 50% depending on the study, was associated with improved survival [30,57].

At our institution, we developed a novel A-B-C-D-E system of classification of preoperative CA 19-9 dynamics based on normalization, direction, and shape of the trajectory of CA 19-9 levels in response to preoperative therapy. Specifically, type A is “always” decreasing to normalization, type B is “bidirectional” with eventual normalization, type C is “consistently” normal, type D is any “decrease” without normalization, and type E is “elevating” without normalization [58].

We studied this classification system in a retrospective analysis of 166 patients with pancreatic cancer who underwent neoadjuvant therapy with at least three CA 19-9 measurements during the treatment period [58]. Within this cohort, CA 19-9 normalization during preoperative therapy was associated with improved postoperative 2-year recurrence-free survival (42.2% vs. 24.3%; *p* = 0.003) and 2-year OS (75.1% vs. 51.3%; *p* = 0.01) rates. Additionally, the direction of the CA 19-9 trajectory during treatment (upwards versus downwards) was associated with survival, and unsurprisingly, the CA 19-9 response type (A, B, C, D, or E) was associated with survival. Patients with type A and B responses had the highest 2-year recurrence-free survival rates (51.2% and 55.6%, respectively) and 2-year OS rates (75.3% and 92.3%, respectively), whereas those with type E responses had the lowest 2-year recurrence-free survival and OS rates (7.1% and 30.8%; both *p* ≤ 0.003). Furthermore, in multivariable analyses to determine whether CA 19-9 normalization alone or our more holistic MD Anderson Type A, B, C, D, E CA 19-9 response classification system better predicted survival outcomes, we found that the CA 19-9 response classification system better predicted recurrence-free survival (*p* < 0.001) and OS (*p* = 0.01) than did CA 19-9 normalization alone [58]. 

CA 19-9 is a useful quantitative tool in assessing the response of PDAC to preoperative therapy, guiding adjustments to treatment regimens, and selecting patients who will ultimately benefit from surgical resection. However, this tool does have limitations (e.g., patients who are CA 19-9 nonproducers, association with benign inflammatory conditions) and should not be used as the sole indicator of response to neoadjuvant therapy.

## 4. Chemotherapy Switch in the Neoadjuvant Setting

Patients with pancreatic cancer treated with neoadjuvant chemotherapy should receive a regimen that is both tolerable and demonstrably effective, as oncologic outcomes are associated with the pathologic treatment response. Multiple studies have shown that major pathologic response is associated with an R0 resection, negative lymph nodes, smaller tumor size, and improved survival [59,60]. 

Thus, for patients who do not tolerate and/or respond to first-line neoadjuvant chemotherapy, it is important to consider a chemotherapy switch (CS). Historically, multidisciplinary teams were apprehensive about CS in the neoadjuvant setting due to the limited data about this treatment approach [61,62]. However, in recent years more institutions have implemented CS and retrospectively published the outcomes [63,64,65]. 

In 2019, our institution published a case series of 25 patients with pancreatic cancer who initially received neoadjuvant FOLFIRINOX but were switched to gemcitabine-abraxane due to poor disease response (64%), poor tolerance of FOLFIRINOX (24%), or a combination of both factors (12%). It should be noted that all patients underwent CS after only 4 cycles of FOLFIRINOX, essentially at the time of first restaging. 21 (84%) of the patients displayed serologic or radiographic responses to the gemcitabine-Abraxane, with 11 (52%) ultimately undergoing curative-intent resection. The OS of the entire cohort was 17 months, with a median OS of 24 months among patients who underwent resection. Without this early preoperative CS, it is expected that these patients would have had worse survival [65]. 

In 2021 the multidisciplinary pancreatic cancer group at the Mayo Clinic retrospectively reviewed 468 patients with locally advanced or BR-PDAC patients treated with neoadjuvant chemotherapy. 329 patients (70%) continued with only their first-line chemotherapy regimen, while 139 (30%) required a CS. Indications for CS included nonmetastatic radiographic progression (42%), biochemical (CA19-9) progression (39%), no objective disease response (25%), and chemotherapy toxicity/intolerance (19.4%), with 38 patients (27%) having multiple indications. Patients who underwent CS did so after a median of 4 cycles of first-line chemotherapy. Within the CS cohort, 100 patients (72%) ultimately underwent curative-intent resection, with no significant differences in RFS (30.0 vs.19.1 months, *p* = 0.13) and OS (41.4 vs. 36.4 months, *p* = 0.94) compared to those who only received first-line chemotherapy prior to resection [63,64]. 

It is imperative to treat patients with a neoadjuvant chemotherapy regimen that is both objectively effective against their disease and clinically tolerable for the individual [64,66,67,68]. As demonstrated in the recent literature, there are a substantial proportion of patients with locally advanced or BR-PDAC that suffer from inadequate radiographic or biochemical response, disease progression, and/or clinical intolerance to first-line regimens. A timely preoperative CS, after 4 cycles of first-line therapy, is a feasible strategy that can facilitate eventual curative-intent resection in said patients and improve OS [63,64,65].

## 5. Implications of Vascular Involvement in PDAC

As described previously, the extent of a tumor’s vascular involvement is a key criterion, and occasionally the only criterion, for determining the stage and resectability of disease [1,3,16,27,37,38]. Unfortunately, only 10–20% of patients have resectable disease at the time of diagnosis. However, vascular resection and reconstruction during tumor resection increase the proportion of patients eligible for curative-intent resection with negative margins [69,70]. 

Historically, vascular involvement was considered a relative contraindication to curative resection of PDAC [71]. However, major advances in radiologic evaluation, surgical technique, patient selection, and multimodality treatment have resulted in improved outcomes (specifically, improved perioperative morbidity, perioperative mortality, margin status, and survival) [29,69,70]. In fact, contemporary data demonstrate comparable survival outcomes among patients who undergo R0 resection with venous reconstruction and those who undergo standard pancreatoduodenectomy (PD) [72]. Additionally, increased use of preoperative therapy for BR-PDAC has expanded the population of patients eligible for surgical resection, including those requiring vascular resection and reconstruction [73,74,75]. 

In 2009, the Americas Hepato-Pancreato-Biliary Association and the Society of Surgical Oncology released a consensus statement regarding surgical treatment of resectable PDAC and BR-PDAC. Afterward, PD with venous resection and reconstruction became the standard of practice for PDAC with venous involvement [75,76]. 

Arterial resection and reconstruction have been controversial because of technical difficulty and prohibitive morbidity and mortality [29,74]. Authors have reported acceptable outcomes when performed on highly selected patients by experienced surgeons [69]. Also, several studies demonstrated favorable outcomes when arterial resection was performed in patients who have undergone multimodality therapy, although it should be noted that the perioperative morbidity rates were often greater than 30% [74,77,78,79]. Centers with increased expertise in arterial resection have reported median OS times ranging from 17 to 20 months [80,81,82,83]. It is imperative to note that arterial involvement is indicative of advanced disease and aggressive tumor biology, and the outcomes reported here are in patients who were carefully selected, treated at high-volume centers, and operated on by surgeons with vast experience in arterial resection and reconstruction.

### 5.1. Venous Resection and Reconstruction

If a pancreatic tumor involves the PV, SMV, or PV-SMV confluence and cannot be separated from the vein segment without leaving residual disease on or within the vein, venous resection and reconstruction should be performed [29,70,76,84]. Types of venous resection and reconstruction include lateral venorrhaphy and primary repair (VR0), tangential resection with a saphenous vein patch (VR1), segmental resection with splenic vein ligation and either a primary anastomosis (VR2) or interposition graft (VR3), and segmental resection with splenic vein preservation and either a primary anastomosis (VR4) or interposition graft (VR5) [29,75,85]. 

Venous reconstructions should be performed with interrupted 5-0 or 6-0 polypropylene (Prolene) sutures [86]. Reconstruction with an autologous vein graft is standard practice; at our center, the left internal jugular vein is preferred owing to technical simplicity and minimal morbidity [29]. Other options for autologous conduits include the greater saphenous vein, superficial femoral vein, left renal vein, and, occasionally, inferior mesenteric vein [87,88]. Prosthetic grafts can be used if a suitable autologous vein is not available but are otherwise not recommended because of risks associated with their use in a contaminated operative field susceptible to a pancreatic fistula [40,86]. However, up to 5 cm of distance can potentially be managed with a primary anastomosis via division of the splenic vein and full mobilization of the liver. This can preclude the need for an interposition graft during venous reconstruction [89,90].

### 5.2. Arterial Resection and Reconstruction

Arterial involvement was previously considered an absolute contraindication for resection. However, patients with <180° arterial abutment and adequate response to neoadjuvant therapy may be considered for resection [27,38,69,91]. In the setting of SMA abutment, the tumor can be divested from the SMA adventitia. However, SMA resection and reconstruction are contraindicated in this setting owing to the prohibitive risk of postoperative morbidity and mortality, including intestinal ischemia and nutritional depletion, because of complete denervation of the midgut. For tumors involving the CA or CHA, arterial resection may be considered. Encasement of a short segment of the CHA can be treated with resection and reconstruction [29]. The Appleby procedure (distal pancreatectomy with CA resection) can be performed in patients with cancer of the pancreatic body or tail involving the celiac trunk. If retrograde arterial inflow from the SMA, pancreatoduodenal arcades, and gastroduodenal artery can support the hepatobiliary system and stomach, CA reconstruction is not required [92,93,94]. 

The Mary Ann and Charles LaBahn Pancreatic Cancer Program at the Medical College of Wisconsin has described a “supercharged” Appleby technique in which a reversed saphenous vein graft is used to augment flow from the divided CA and CHA. The rationale for using this procedure is that it (1) maximizes hepatic and gastric perfusion, especially if the left gastric artery is taken with the CA, and (2) restores normal arterial flow, which may prevent complications such as hepatic abscess and delayed gastric emptying [74]. While there is insufficient data to support performing it routinely, the “supercharged” Appleby technique can be used, if necessary, based on concerns about adequate arterial flow.

### 5.3. Outcomes of Vascular Resection and Reconstruction

Several studies have demonstrated no differences in 30-day mortality, in-hospital mortality, or perioperative morbidity rates among patients undergoing PD with venous resection and reconstruction and patients undergoing PD alone [81,84,95,96,97,98,99,100,101]. Furthermore, patients undergoing PD with venous resection and those undergoing PD alone have similar survival [84], and PD with venous resection is considered safe when performed by experienced surgeons for appropriately selected patients at high-volume centers [69,70,102]. Additionally, data on venous resection in combination with any type of pancreatectomy have demonstrated similar long-term survival for those undergoing surgery with and without venous resection [103,104]. A 2012 meta-analysis demonstrated no differences in perioperative morbidity, mortality, or 5-year OS between patients who underwent venous resection and those who did not [105]. 

Post-operative thrombosis following vascular reconstruction is a known but rare complication in the acute postoperative setting, with researchers in one study reporting a 90% patency rate 1 year after surgery [86]. Although thrombosis can develop over time, most studies have had long-term patency rates greater than 80% [84,86,106]. In our 2018 series of 120 patients who underwent PD with PV resection, we observed no association between the extent of venous resection or reconstruction and the risk of PV thrombosis. Additionally, thrombosis appeared to be more representative of tumor biology than technical issues associated with reconstruction [107]. 

Currently, there are no guidelines regarding the optimal pharmacologic regimen for patients undergoing pancreatectomy with vascular resection and reconstruction [74]. At our institution, patients are treated with aspirin and prophylactic enoxaparin (Lovenox) beginning in the immediate postoperative period. Patients receive prophylactic Lovenox for 28 days after resection and continue taking aspirin indefinitely.

In summary, concomitant vascular resection and reconstruction increase the degree of complexity of pancreatectomy for PDAC. Pancreatic surgeons therefore must gain a comprehensive understanding of the diagnostics, optimization, and surgical techniques involved in PD with vascular resection and carefully consider appropriate patient selection for said operations.

## 6. Prehabilitation and Medical Optimization

Although preoperative therapy should be considered for patients with anatomically resectable PDAC and is the standard of care for BR-PDAC and locally advanced or unresectable PDAC, it is associated with adverse effects and toxicity. Preemptive, proactive management of these effects is critical both to prevent impairment of a patient’s fitness and to preserve the patient’s quality of life during therapy. Additionally, neoadjuvant therapy can provide time for implementation of prehabilitation programs designed to optimize patient nutrition, therapy tolerance, and, ultimately, functional status prior to surgical resection [108]. 

More than 80% of patients with pancreatic cancer suffer from cancer-induced unintentional weight loss, malnutrition, and/or exocrine pancreatic insufficiency, all of which are associated with poor performance status and survival [109,110,111]. Unfortunately, neoadjuvant therapy has multiple adverse effects that can worsen these conditions, including nausea, vomiting, diarrhea, oral ulceration, xerostomia, indigestion, and alteration of intestinal motility [112,113]. If not adequately managed, these effects can adversely affect the completion of preoperative therapy or even prevent surgical resection [114,115,116]. 

In addition to managing the adverse effects and toxicity of therapy, intentionally identifying opportunities to improve patient fitness and eligibility for surgery during neoadjuvant treatment is important. Several recent studies demonstrated the feasibility and effectiveness of prehabilitation and nutritional interventions in the preoperative therapy period, resulting in intentional weight adjustments when appropriate, decreased malnutrition, improved performance status, and, ultimately, improved postoperative outcomes [108,117]. In 2018, a prospective randomized controlled trial of nutritional intervention (560 kcal/day eicosapentaenoic acid–enriched nutritional supplements) in patients undergoing neoadjuvant therapy demonstrated an increased psoas major muscle area ratio with the intervention [118]. In another study of 109 patients with pancreatic cancer who received nutritional intervention prior to resection, nutritional support provided within 3 months after diagnosis was a significant predictor of better survival (30% absolute difference in 2-year OS compared to those who received nutritional intervention over 3 months after diagnosis) [117]. The International Study Group of Pancreatic Surgery strongly encourages consultation and follow-up with a nutritionist or dietitian for patients during the preoperative therapy period [112].

The concept of prehabilitation is not limited to nutrition but should also focus on improving cardiorespiratory function [119]. Studies investigating the effectiveness of prehabilitation with thoracic and abdominal surgery demonstrated improvements in short-term postoperative outcomes following preoperative aerobic conditioning and strength training [120,121]. Another study demonstrated reduced length of stay and pulmonary morbidity among patients who underwent prehabilitation prior to pancreas surgery when compared with a historical control group [122]. Other studies of prehabilitation in patients undergoing PD or hepatectomy have demonstrated decreased length of stay, reduced weight loss, and improved albumin levels in the prehabilitation cohorts [123]. 

Between 2015–2017 we conducted a prospective, single-arm trial at MD Anderson investigating relationships between physical activity and both changes in physical function and health-related quality of life (HRQOL) amongst patients with resectable PDAC enrolled in a home-based prehabilitation program that place throughout pre-operative therapy (chemotherapy, CRT, or both sequentially) until pre-operative surgical evaluation. Participants were advised to participate in at least 60 min per week of moderate-intensity aerobic exercise and at least 60 min per week of full-body strengthening exercises. Additionally, all participants met with a registered dietician who provided individualized nutrition recommendations. Patients had a statistically (all *p* < 0.05) and clinically significant improvement in submaximal exercise capacity as measured by the six-minute walk test, improvement in leg strength as measured by the five times sit-to-stand test, and their gait speed as measured by the three-meter walk test [124]. In cancer patients, these tests have been shown to be predictors of treatment complications, post-operative complications, activities of daily living, and even survival [125,126,127,128,129,130]. Furthermore, physical activity was positively associated with improved physical function and HRQOL (as measured by the Functional Assessment of Cancer Therapy–Hepatobiliary questionnaire). This pragmatic, prospective trial illustrates the importance, benefits, and feasibility of a prehabilitation program during preoperative treatment for PDAC [124]. 

We subsequently conducted a randomized controlled trial between 2017–2021, the PancFit randomized clinical trial. 151 patients undergoing preoperative therapy were randomized to either Arm A (enhanced usual care) or Arm B (prescribed aerobic and resistance exercise during pre-operative) therapy. All participants were encouraged to be physically active and received activity trackers. Participants randomized to Arm A received a handout on the benefits of exercise, a stretching guide, and a nutrition guide. They did not receive a specific exercise prescription. Participants randomized to Arm B were prescribed the exercise program implemented in the prospective, single-arm trial discussed above. Participants in both arms had statistically and clinically significant improvements in the six-minute walk test, the primary outcome of this study, as well as improvement in other functional measures of upper body and lower body function. While the improvements in these measures did not differ significantly between the two arms, a pooled analysis of all participants found that moderate-to-strenuous activity was associated with a higher likelihood of resection and a lower likelihood of readmission after resection. This trial further highlights the benefits of encouraging pre-operative physical activity and the feasibility of a prescribed rehabilitation program in patients with pancreatic cancer preparing for resection [131]. 

Unfortunately, the data on prehabilitation with surgical resection for PDAC remains limited, indicating the need for additional prospective clinical trials. However, these data are promising and convey a benefit in terms of nutrition, performance status, and, ultimately, outcomes in patients participating in rehabilitation programs.

## 7. Neoadjuvant Therapy and Postoperative Morbidity

Pancreatic resection, especially PD, can result in major postoperative complications, including pancreatic fistulae, hemorrhage, delayed gastric emptying, and sepsis [132,133]. Rates of postoperative complications range from 15% to 45% [134,135,136,137], and these complications can lead to delayed or failed initiation or completion of adjuvant therapy, with researchers in several studies reporting up to 47% of patients being unable to initiate or complete adjuvant therapy [5,138,139,140,141,142,143,144]. A retrospective study of the Surveillance, Epidemiology, and End Results Program Medicare database identified 2440 patients 65 years of age or older who underwent upfront resection of PDAC and demonstrated that only 35% of Medicare patients received any adjuvant chemotherapy and only 7% completed their recommended treatment courses [14]. 

Despite early concerns that preoperative therapy would increase postoperative complication rates, recent studies and meta-analyses have consistently demonstrated no difference in morbidity between patients given neoadjuvant therapy and those who undergo upfront surgery [145]. A systemic review and meta-analysis performed in 2020 that comprised 45 studies of 1904 patients who underwent neoadjuvant therapy and 1455 who underwent upfront resection identified no difference in postoperative mortality or morbidity rates between the two groups. This reported evidence conclusively demonstrates that neoadjuvant therapy does not adversely affect perioperative outcomes in appropriately selected patients who undergo treatment by experienced surgical teams [146].

## 8. Future Directions

The role of neoadjuvant therapy in the treatment of PDAC continues to expand as evidenced by increased data, ongoing and emerging clinical trials, and growing enthusiasm for it in the surgical and oncology communities. Surgeons and multidisciplinary teams should carefully consider several factors prior to initiation of preoperative therapy for PDAC, including tumor resectability, vascular involvement, and patient optimization. Additionally, as cancer care becomes more personalized, a deeper understanding of an individual patient’s tumor biology will be of paramount importance for personalizing preoperative therapy regimens and appropriately adjusting them as needed [1,65,147]. 

In the future, we anticipate the development of improved mechanisms for monitoring disease response to preoperative therapy. Currently, CA 19-9 is the only reliable biomarker of response to therapy [1,16,38,53,148]. However, up to 10% of patients with pancreatic cancer are CA 19-9 nonproducers and one-third have normal baseline CA 19-9 levels at presentation, limiting assessment of response in these patients [37,55,149,150]. Although the standard Response Evaluation Criteria in Solid Tumors can adequately indicate the effectiveness of the treatment of most cancers, their utility in assessing the response of PDAC to therapy is limited. Several studies demonstrated that after neoadjuvant therapy for PDAC, the radiographic response did not accurately predict tumor unresectability [23,151,152]. 

We also anticipate that future methods of assessing disease response to therapy that are more dynamic than current modalities will improve the delivery of appropriate, effective therapy. Several ways to examine the response to therapy that are currently under exploration include cell-free DNA or circulating tumor DNA, systemic inflammatory indexes, assessing metabolic response via positron emission tomography (PET), and radiomics. 

Several studies have examined the use of circulating tumor DNA to gauge the therapeutic response of PDAC, with one prospective trial demonstrating that detectable circulating tumor DNA after tumor resection was associated with worse recurrence-free survival (5 months vs. 17 months) and OS (11 months vs. not reached) than those with no detected circulating tumor DNA [153]. More recent reports described the use of exosome-derived DNA, which arises from viable pancreatic cancer cells, demonstrating that increased occurrence of KRAS mutations in exosome-derived DNA was a predictor of progression-free survival and OS and correlated with disease progression [154,155]. The results of these studies suggest that circulating tumor DNA and/or exosome-derived DNA can be used to provide dynamic information about PDAC response to neoadjuvant therapy.

The systemic immune-inflammation index (SIII), an easily obtainable biomarker reflecting the extent of systemic inflammation, has been shown to be an independent predictor of cancer-specific survival, and recurrence in patients with resectable pancreatic cancer [156]. A group of Dutch scientists published a study evaluating the prognostic value of SIII in patients with advanced PDAC treated with FOLFIRINOX or FOLRINIOX followed by SBRT. This study demonstrated that increases in SIII during treatment were associated with poor survival, and that further work is warranted to clarify the role of SIII in assessing treatment response in patients with pancreatic cancer [157].

Multiple retrospective studies at MD Anderson have demonstrated that high-delta PDAC tumors (those with a conspicuous border) on CT images are associated with multiple pathway mutations, increased potential for metastasis, and worse clinical outcomes when compared with low-delta tumors (those with inconspicuous borders) [158,159]. A prospective trial at MD Anderson compared the following clinical outcomes of PDAC based on radiomic responses to preoperative therapy: type I, in which the tumor interface becomes or remains well defined, and type II, in which the tumor interface becomes less defined. The investigators found that patients with low q-delta tumors had better OS and progression-free survival than those with high q-delta tumors, validating the findings of the retrospective studies. Additionally, regardless of the delta category, patients with type I responses had better OS and progression-free survival than those with type II responses. Lastly, all type II responders had R1 resection margins, whereas all type I responders had R0 resection margins [160]. 

Regarding the use of PET, current NCCN guidelines only state that it may be considered after formal pancreatic CT protocol in high-risk patients to detect extra-pancreatic metastases [27]. In 2022, the multidisciplinary pancreatic cancer group at the Mayo Clinic evaluated their institution’s experience with preoperative metabolic imaging (18F-fluorodeoxyglucose-PET [FDG-PET]) to predict neoadjuvant therapy response and survival in patients with BR-PDAC. They found that after therapy, the metabolic response was the single largest independent preoperative predictor of pathologic response and survival, even superior to preoperative biochemical response [152]. Additionally, several meta-analyses of retrospective studies also demonstrated the utility of metabolic response in predicting the pathologic response of PDAC after neoadjuvant therapy [161,162]. Given the association between FDG-PET findings and post-neoadjuvant therapy pathologic response and survival, it could potentially serve as an assessment of response to preoperative therapy that guides further treatment (curative intent resection, continuation of systemic therapy, chemotherapy switch, etc.) However, given that much of this evidence is from heterogeneous retrospective studies, standardized prospective clinical trials investigating the use of FDG-PET in patients with pancreatic cancer are warranted [152,161,162].

## 9. Conclusions

In summary, the benefit of neoadjuvant therapy is not limited to improving the likelihood of resection in patients with BR-PDAC. Neoadjuvant therapy enhances patient selection for surgery, provides time for prehabilitation that can reduce perioperative complications, and ensures delivery of systemic therapy for PDAC. These benefits can increase the quantity and quality of life for a plethora of patients with pancreatic cancer, including those who do not undergo resection after careful selection. As further evidence regarding germline mutations and somatic profiling of pancreatic cancer becomes available, opportunities to better tailor preoperative therapy to improve patient outcomes will increase. Until there is more data supporting a personalized approach to neoadjuvant therapy for PDAC, a comprehensive understanding of the key surgical considerations of resectability, management of vascular involvement, and patient optimization is critical for guiding treatment sequencing and decision-making.

## Figures and Tables

**Table 1:** NCCN criteria for PDAC resectability status at diagnosis based on pancreatic CT.

Resectability	Arterial Involvement	Venous Involvement
Resectable	No tumor contact with major arterial structures (CA, SMA, and/or CHA)	No tumor contact with SMV or PV ≤180° contact WITHOUT vein contour irregularity
Borderline Resectable	Pancreatic head/uncinate process: Solid tumor contact with CHA without extension to CA or hepatic artery bifurcation Solid tumor contact with the SMA of ≤180° Solid tumor contact with variant arterial anatomy (example: accessory right hepatic artery, replaced right hepatic artery, replaced CHA, etc.) Pancreatic body/tail: Solid tumor contact with the CA of ≤180°	Solid tumor contact with the SMV or PV of >180° ≤180° solid tumor contact with contour irregularity of the vein or thrombosis of the vein BUT with suitable vessel proximal and distal to the site of involvement, allowing for adequate vein resection and reconstruction Solid tumor contact with the inferior vena cava
Locally Advanced	Pancreatic head/uncinate process: Solid tumor contact >180° with the SMA or CA Pancreatic body/tail: Solid tumor contact of >180° with the SMA or CA Solid tumor contact with the CA and aortic involvement	Unreconstructible SMV or PV due to extensive tumor involvement or venous occlusion

Note: all recommendations are category 2A (uniform NCCN consensus that the intervention is appropriate based on lower-level evidence) [27].

**Table 2:** The MD Anderson resectability criteria for PDAC [1].

Factors	Potentially Resectable	Borderline Resectable	Unresectable
Tumor Anatomy (A)	No radiographic tumor interface with CA, SMA, or CHA No interface with SMV or PV or <180° interface without vein contour irregularity	Tumor-vessel circumferential interface <180° with SMA or CA Reconstructable short-segment interface with CHA Interface with SMV or PV >180° and/or reconstructable occlusion	>180° radiographic interface with CA or SMA Unreconstructable SMV or PV due to tumor involvement or occlusion
Tumor Biology (B)	No clear evidence of distant metastatic disease No evidence of regional lymphadenopathy CA 19-9 level normal or only mildly elevated	Imaging findings suggestive but not diagnostic of metastatic disease OR Confirmed regional lymphadenopathy OR CA 19-9 level moderately elevated	Confirmed extraregional lymphadenopathy
Patient Condition (C)	Good performance status (ECOG 0–1) No major comorbidities	Suboptimal performance status (ECOG 2–3) Multiple comorbidities with capacity for optimization and prehabilitation	Poor performance status (ECOG~3 or higher) Poor comorbidity profile with no capacity for optimization

ECOG, Eastern Cooperative Oncology Group.

**Table 3:** The International Association of Pancreatology consensus definition of BR-PDAC and its specific criteria [38].

**Anatomic (BR-A)**	If SMV/PV involvement only, then tumor contact ≥180° or bilateral narrowing/occlusion not exceeding the inferior border of the duodenum If arterial involvement: SMA, CA: tumor contact of <180° without evident deformity/stenosis CHA: tumor contact without evident tumor contact with the PHA and/or CA
**Biologic (BR-B)**	Tumor potentially resectable anatomically with clinical findings suspicious for, but not proven, distant metastasis, including: CA 19-9 level > 500 U/mL Regional lymph node metastasis diagnosed via biopsy or PET-CT
**Patient Condition (BR-C)**	Anatomically resectable PDAC and ECOG score of ≥2

PHA, proper hepatic artery; PET, positron emission tomography.
